# Demonstrating the sustainability of capacity strengthening amidst COVID-19

**DOI:** 10.1093/inthealth/ihab004

**Published:** 2021-02-16

**Authors:** P Abomo, E M Miaka, S J Crossman, A Hope

**Affiliations:** Centre for Capacity Research, Liverpool School of Tropical Medicine, 2 rue paul gauguin, 78340, Les Clayes Sous Bois, France; Programme National de Lutte contre la Trypanosomiase Humaine Africaine, Avenue de la Liberation, Kinshasa, DRC, UK; Centre for Capacity Research, Liverpool School of Tropical Medicine, Pembroke Place, Liverpool L3 5QA, UK; Vector Biology Group, Liverpool School of Tropical Medicine, Pembroke Place, Liverpool L3 5QA, UK

**Keywords:** capacity strengthening, COVID-19, DRC, Gambian human African trypanosomiasis, lockdown, tiny targets

## Abstract

The global disruptions caused by the coronavirus disease 2019 crisis posed a threat to the momentum the vector control team at the Liverpool School of Tropical Medicine (LSTM) and the Programme National de Lutte contre la Tryaponosomiase Humaine Africaine (PNLTHA) had built in their efforts to control tsetse fly populations in the Democratic Republic of Congo. But despite the pandemic and global lockdown, field activities did continue and the same impressive results in tsetse fly reduction were observed and the team followed this by completing a round of ‘tiny target’ deployment without any external presence. Such a success was possible due to the investment in vector control capacity strengthening undertaken by the LSTM and PNLTHA.

## Introduction

The coronavirus disease 2019 (COVID-19) pandemic has had an unprecedented impact around the world with health systems struggling to cope. The pandemic has the potential to inflict severe disruption in low- and middle-income countries (LMICs) where health systems are already fragile and it will inevitably impact on neglected tropical disease (NTD) programmes.^1^ For many NTDs, 2020 was an important year, with several elimination targets to be reached as described in the 2012 World Health Organization (WHO) NTD road map.^2^ In response to the pandemic, the WHO advised that community-based surveys, mass drug administration and active case finding for NTDs should be temporarily suspended but recommended that vector control activities continue.^3^ Despite the challenges being faced, we report on some positive developments in vector control from the Democratic Republic of Congo (DRC).

Historically, vector control for Gambian human African trypanosomiasis (GHAT) did not form a major part of efforts to control the disease due to the costs and logistics associated with available technologies. Consequently the disease was controlled primarily by large-scale screening and treatment. That situation changed with the development of ‘tiny targets’, small blue and black screens impregnated with insecticide that attract and kill tsetse flies. The technology is in use in Chad, Côte d'Ivoire, Guinea and Uganda and has been shown to significantly reduce tsetse populations and disease incidence.^4^ In 2015, tiny targets were introduced to the DRC through a pilot project between the Liverpool School of Tropical Medicine (LSTM) and the Programme National de Lutte contre la Tryaponosomiase Humaine Africaine (PNLTHA). Working initially in the health zone of Yasa Bonga in the former Province of Bandundu, tiny targets were used to reduce tsetse densities by >85%,^5^ leading to a scale-up of vector control activities to complement medical interventions in 11 health zones, increasing coverage from approximately 2000 km^2^ to approximately 12 000 km^2^.

Prior to the introduction of tiny targets, vector control had not been done systematically in the DRC and the PNLTHA had only two vector control staff, both based in Kinshasa. In the initial pilot study, all field activities were coordinated by the LSTM and one member of PNLTHA staff. To scale-up tsetse control, and to have a sustainable vector programme, the need to strengthen capacity for vector control within the PNLTHA was recognized. A capacity gap analysis was conducted by the Centre for Capacity Research at the LSTM and a capacity strengthening action plan was developed, including actions to strengthen ownership of the tiny target programme by the PNLTHA and involvement of province and health zone levels, staff development and recruitment and to update equipment and operational materials.

## COVID lockdown

Since 2019, vector control activities expanded, with activities implemented in five health zones in two provinces, but progress was threatened by COVID-19. National lockdown policies prevented LSTM staff from travelling to the DRC to support activities and, in the DRC, restrictions made it impossible for the Kinshasa PNLTHA entomologists to support the newly recruited provincial teams. However, even though the provincial-level teams had never implemented activities without support from either the central level of the PNLTHA or from the LSTM, they managed to continue the planned implementation of entomological monitoring in June. To aid their progress, the LSTM and PNLTHA provided remote training on the use of a phone app designed for data collection, storage and monitoring.

Movement restrictions have now eased in the DRC and travel to the field is possible and the second deployment of tiny targets for the year has been completed in five health zones. In one health zone the deployment was implemented without the presence of staff from the central level in Kinshasa—the first time that a deployment was locally managed. In the remaining four health zones, the deployments were completed with support from the PNLTHA Kinshasa. This is the largest deployment of tiny targets that has been implemented in the DRC without external presence, with approximately 23 500 tiny targets deployed using traditional canoes or by foot over approximately 4700 km^2^.

## What can we learn from this experience?

Capacity strengthening for vector control has been crucial for the continuance of vector control in the DRC. It demonstrated the potential for empowerment and autonomy of local teams to produce a lasting impact and the sustainability of global health interventions.^6,7^ If the pandemic had occurred 2 y earlier, it is likely that the impact on tsetse fly control operations in the DRC would have been far more drastic, as there was no vector control capacity outside of Kinshasa. It is due to capacity strengthening that vector control in the DRC is on its way to a much more sustainable system thanks to the ongoing work to transition responsibilities to provincial and health zone levels.

As observed during the Ebola outbreak in Guinea, the COVID-19 pandemic has demonstrated the resilience of tsetse fly control in a crisis. In the Ebola outbreak, GHAT medical activities had to be suspended but tiny target deployment continued and was shown to have a significant impact on disease incidence.^8^ The example from Guinea highlights the importance of continuing vector control during crises when medical surveys must be put on hold. As a result of our capacity strengthening work, vector control has been maintained during the COVID-19 pandemic, as recommended by the WHO,^3^ providing vital protection to local communities.

## Data Availability

The data that support the findings of this study are available from the corresponding author, [P.A.], upon reasonable request.
